# p53 CRISPR Deletion Affects DNA Structure and Nuclear Architecture

**DOI:** 10.3390/jcm9020598

**Published:** 2020-02-22

**Authors:** Aline Rangel-Pozzo, Samuel Booth, Pak Lok Ivan Yu, Madhurendra Singh, Galina Selivanova, Sabine Mai

**Affiliations:** 1 Cell Biology, Research Institute of Oncology and Hematology, University of Manitoba, CancerCare Manitoba, Winnipeg, MB R3C 2B1, Canada; samuel.booth@gmail.com (S.B.); ivanyu2@live.ca; (P.L.I.Y.); 2 University of British Columbia, Vancouver, BC V6T 1Z4, Canada; 3 Department of Microbiology, Tumor and Cell Biology, Karolinska Institute, Stockholm SE-171 76, Sweden; madhurendra.singh@ki.se; (M.S.); galina.selivanova@ki.se; (G.S.)

**Keywords:** p53, nuclear architecture, DNA structure, telomeres, 3D-Imaging, super-resolution microscopy

## Abstract

The *TP53* gene is a key tumor suppressor. Although the tumor suppressor p53 was one of the first to be characterized as a transcription factor, with its main function potentiated by its interaction with DNA, there are still many unresolved questions about its mechanism of action. Here, we demonstrate a novel role for p53 in the maintenance of nuclear architecture of cells. Using three-dimensional (3D) imaging and spectral karyotyping, as well as super resolution microscopy of DNA structure, we observe significant differences in 3D telomere signatures, DNA structure and DNA-poor spaces as well gains or losses of chromosomes, between normal and tumor cells with CRISPR (Clustered Regularly Interspaced Short Palindromic Repeats)-deleted or wild-type *TP53*. Additionally, treatment with Nutlin-3 results in differences in nuclear architecture of telomeres in wild-type but not in p53 knockout MCF-7 (Michigan Cancer Foundation-7) cells. Nutlin-3 binds to the p53-binding pocket of mouse double minute 2 (MDM2) and blocks the p53-MDM2 interaction. Moreover, we demonstrate that another p53 stabilizing small molecule, RITA (reactivation of p53 and induction of tumor cell apoptosis), also induces changes in 3D DNA structure, apparently in a p53 independent manner. These results implicate p53 activity in regulating nuclear organization and, additionally, highlight the divergent effects of the p53 targeting compounds Nutlin-3 and RITA.

## 1. Introduction

The tumor suppressor p53 is a transcription factor that is able to induce a wide variety of signaling pathways leading to cell cycle arrest, angiogenesis, apoptosis, DNA repair and cell senescence, among others [1,2,3,4,5,6,7,8,9,10]. Its crucial role, realized through its interactions with other proteins and DNA, is the protection of cells from malignant transformation [1,2,3,4,5,6,7,8,9,10,11,12,13]. Canonically, signals induced by cellular stress lead to the accumulation of p53 in the nucleus via a post-translational mechanism and, consequently, the expression of p53-activated genes related to cell-cycle arrest, senescence and apoptosis [14,15]. However, by binding to B-cell lymphoma 2 (BCL)-2 family proteins, p53 can enhance apoptosis in a transcription-independent manner [14,15]. p53 activity is also regulated post-translationally by mouse double minute 2 (MDM2), an E3-ubiquitin ligase which blocks p53 transactivation and marks p53 for proteasome degradation [14]. As a result, p53 activity decreases in resting cells until a stress signal stimulates a rapid stabilization of the protein. p53 has been linked to a host of tumor suppressing activities [16,17] and since many antitumor pathways are initiated by p53, its function is commonly inhibited in cancers [18]. Approximately 50% of tumors exhibit p53 mutations, including non-sense (p53-null) or missense mutations (stable mutant proteins) [19]. In tumors that express wild-type p53, p53 function is usually abrogated through other mechanisms, such as overexpression of MDM2 [20]. Inactivation of p53 through mutation or dysregulation of the p53 regulatory pathway is an essential step in tumor development [1,2,3,4,5,6,7,8,9,10,11,12,13]. 

The prevalence of p53 mutations in cancer and the wide range of p53 antitumor activities make p53 a promising target for anticancer therapies. Therefore, efforts have been made to identify low molecular weight compounds that can pharmacologically reactivate p53 [21]. In tumors expressing wild-type p53, targeting the auto regulatory interaction between MDM2 and p53 can significantly stabilize p53 protein levels and induce apoptosis. Vassilev et al. (2004) demonstrated that the small molecule Nutlin-3 binds to the p53-binding pocket of MDM2 and blocks the p53-MDM2 interaction [22]. Nutlin-3 induces p53 dependent growth arrest and apoptosis in human tumor xenografts in nude mice [22]. The small molecule RITA also blocks p53-MDM2 interaction and promotes activation of p53 dependent anti-tumor effects both in vitro and in vivo [23]. Interestingly, although both compounds target the p53—MDM2 interaction, the effects on p53 activity are different [24]. Nutlin-3 treatment results in the upregulation of cell cycle regulators, whereas RITA (named for Reactivation of p53 and Induction of Tumor cell Apoptosis) activates p53-regulated apoptosis-related genes [24]. RITA can also induce cell death in p53-defective cells independently of p53 function via activation of c-Jun N-terminal kinases (JNKs), also referred to as stress-activated kinses (SAPKs)and p38, for instance [25]. 

In light of the expanding spectrum of p53 anti-tumor activity, it is essential to investigate the relation of p53 to other components of cancer. Changes in nuclear architecture are a key hallmark of cancer [26,27,28,29,30,31,32,33]. The three-dimensional (3D) nuclear organization can be described by the 3D organization of chromosomes, where chromosomes occupy specific nuclear areas organized into discrete chromosome territories, with DNA interactions across boundaries infrequent [34,35,36]. Co-expressed genes are spatially clustered within the nucleus and disruption of physical gene contacts abrogates their co-expression, confirming the requirement of gene contacts for their co-regulation [37]. Additionally, chromatin organization is crucial for regulating gene expression, genome maintenance and repair [37,38,39]. Therefore, many studies have implicated changes in the nuclear architecture as a component for neoplastic transformation and genomic instability [40,41,42,43,44,45].

3D telomere signatures are a useful method for studying architectural changes in the nucleus of malignant and non-transformed cells [27,28,29,30]. Telomeres consist of (TTAGGG)_n_ repeats that are present at the ends of all eukaryotic chromosomes and form unique nucleoprotein complexes [46]. Telomeres are essential for protecting the ends of chromosomes from DNA damage responses that can result in chromosomal fusions and genetic alterations [47]. Telomeres, like chromosomes, also exhibit a proper spatial distribution and organization [48]. It has been demonstrated that normal lymphocytes telomeres are organized in a non-overlapping manner and their localization during the cell cycle is tightly regulated [48]. Dysregulation in the normal distribution of telomeres is an early event in tumorigenesis [27,28,29,30]. Quantitative 3D telomere fluorescence in-situ hybridization (3D Q-FISH) has previously been used to characterize telomere dysfunction, changes in the nuclear organization and genomic instability [27,28,29,30,40,41,42,43,44,45]. For example, the presence of telomere clusters (telomere aggregates) provides an indication of telomeric fusions and chromosomal instability [42]. Telomere signals can be characterized in terms of 3D distribution and intensity, since intensity is proportional to telomere length [49]. These telomeric features can be analyzed and quantified from 3D images of fixed cell nuclei using TeloView^TM^ (Materials and Methods) [48,49,50]. Thus, 3D imaging and quantitative 3D analysis of telomeres can provide additional information about the global nuclear architecture of cells. Additionally, 3D telomere profiling has been shown to be successful in stratifying patients and predicting response to therapy [28]. The analysis of more than 3000 tumors had shown that p53 mutations are strongly correlated with a high genomic instability [51,52,53]. Ectopic expression of three of the most frequent p53 mutants in colon cancer cells was sufficient to induce remodeling of the nuclear architecture of telomeres and chromosomal rearrangements [51,52,53].

Studies in Drosophila have provided evidence that p53 can form long-range interactions with multiple targets within a large genomic region (>300 kb) [53]. Link et al. (2013), using two-dimensional (2D) images, observed that the chromatin configuration was not affected by p53 status or DNA damage [54]. This observation supports the idea of ‘pre-programmed’ chromatin architecture [53,54]. In human cells, Mello et al. (2013) mapped the interaction between distal p53-activated enhancers and multiple target loci and suggested that chromatin loops formation was not dependent on p53 [55]. However, no study has been performed using high-resolution microscopy to evaluate whether p53 tumor suppression function could affect higher-order chromatin organization.

In the current study, our aim was to investigate changes in the nuclear architecture in a tumor cell line, which expresses wild-type p53, and an isogenic cell line in which p53 was deleted. Additionally, the cells were treated with the p53-activating compounds Nutlin-3 and RITA to observe the effects of p53 anti-tumor activity on the nuclear architecture of these cells. We used 3D telomere profiling to examine changes in nuclear architecture in p53-knockout cells and in cells that have pharmacologically activated p53 activity with RITA or Nutlin-3. 3D Structured Illumination Microscopy (3D-SIM) was used to observe nanoscale changes in DNA structure, because nanoscale chromatin structure cannot be resolved by conventional widefield microscopy due to the diffraction limit of conventional microscopy techniques, which peaks at about 200 nm resolution. 3D-SIM can offer the ability to capture resolutions up to 100 nm resolution in the *x-* and *y-* axes and 200–250 nm in the *z-*axis, which can reveal structural information much closer to the scale of higher order DNA structures within the nucleus [56,57]. This technique has previously been used to quantify broad scale changes in DNA ultrastructure that occur after malignant transformation in cancers [58,59,60]. 3D-SIM technology can be used to capture the size and intensity of fluorescent DNA signals in three dimensions in nuclei stained with DAPI (4′,6 diamidino-2-phenylindole), revealing the size distribution of DNA structures and DNA-poor spaces in super-resolution imaging. The granulometry program calculates the cumulative distribution of micron and submicron DNA “granules” within the cell nucleus, as resolved by 3D-SIM technology [58,59,60]. Previous analysis has shown that coarser chromatin texture is associated with dysregulated nuclear architecture in cancer when compared to non-transformed cells [58,59,60]. The current work assesses the effect of p53 knockout and pharmacological reactivation of p53 on DNA structure in a quantitative manner. Here, we present data linking p53 activity to changes in the nuclear architecture of MCF7 breast cancer cells using the techniques of 3D telomere analysis with TeloView^TM^ and granulometry analysis of super-resolution captured images of DNA structure. We show significant differences between wild-type and p53 knockout cell lines, as well as in cells with pharmacologically enhanced p53 activity.

## 2. Materials and Methods

### 2.1. Tissue Culture and Nutlin-3 and RITA Treatments

The human mammary gland tumor cell line MCF7 was chosen as a representative tumor cell line expressing wild-type p53. A stable p53 knockout line was generated using CRISPR/Cas9 mediated gene deletion as described [61]. A Cas9 expressing vector and a px330 vector coding for a sgRNA targeting exon 3 of p53 were kindly provided by V. Grinkevich (Welcome Trust Sanger institute, Cambridge, UK). Briefly, MCF7 cells stably expressing Cas9 were established, then those cells were transfected with p53 sgRNA. Enrichment of p53-deleted MCF7 cells was performed by Nutlin-3 treatment (10 µM) for 3 days. Deletion of p53 was confirmed by Western blot (Supplementary Figure S1). Wild type and p53 knockout MCF7 cell lines were grown in DMEM (Dulbecco´s Modified Eagle Medium) media (Gibco, Carlsbad, CA, USA.) supplemented with 1X penicillin/streptomycin (Gibco, Carlsbad, CA, USA) and 10% calf serum. The MCF7 cells were edited using CRISPR/Cpf1 mediated gene deletion. The p53 gRNAs used targeted exon 4 (CTGACTCAGAGGGGGCTCGACGC) and exon 9 (AGGTGCGTGTTTGTGCCTGTCCT) of *TP53*, as described by Zetsche et al. (2017) [62]. Cells were seeded onto slides in 4-well 5 mL plates prior to treatment. Cells were incubated with 10 uM Nutlin-3 (Sigma-Aldrich, St. Louis, MO, USA.) and RITA (Cayman chemical, Ann Arbor, MC, USA.) dissolved in dimethyl sulfoxide (DMSO), with equivalent volumes of DMSO being used for vehicle controls. Cells were harvested at 0, 5 and 10 h post-treatment and fixed in 3.7% formaldehyde/1X PBS. Three replicates were performed for each experiment. Fixed cells were stored at 4 °C. Human normal mammary epithelial cells (HMECs) were also obtained for use as a control from the American Type Culture Collection (ATCC PCS-600-010). The cells were thawed and grown as per the distributor’s instructions in Human Mammary Epithelial Cell Basal Medium (ATCC, Manassas, VA, USA), supplemented with Human Mammary Epithelial Cell Growth Kit (ATCC, Manassas, VA, USA) and 1% Penicillin-Streptomycin (Gibco, Carlsbad, CA, USA).

### 2.2. Western Blot Analysis

The total cell lysates extraction and western blot were performed according to standard procedure. The antibodies used were Anti-p53 (sc-126, Santa Cruz Biotechnology, Santa Cruz, CA, USA) and Anti-β-actin monoclonal antibody (Millipore, Burlington, MA, USA) was used as loading control. The horseradish peroxidase-coupled secondary antibodies (Jackson ImmunoResearch, West Grove, PA, USA) and SuperSignal™ West Dura Extended Duration Substrate detection system (Thermo Fischer Scientific, Waltham, MA, USA), and images were taken using ChemiDoc Imaging System (Bio-Rad).

### 2.3. Three-Dimensional Structured Illumination Microsopy (3D-SIM) Slide Preparation

For super-resolution microscopy, cells were grown to 70–80% confluency on 18 × 18 mm high performance coverslips (thickness 1 1/2, 0.170+/-0.005 mm) (Zeiss, Toronto, ON, Canada). The coverslips were fixed for 20 min in a 3.7% formaldehyde solution. After three washes with 1× phosphate-buffered saline (PBS), the cells were permeabilized by a 10-min incubation in a 0.5% Triton-100X solution. Samples were stained with 5 µL of 10 µg/mL 4,6-diamidino-2-phenylindole (DAPI) and incubated overnight in the dark at 37 °C. Excess DAPI was rinsed with water. The coverslips were mounted on slides with 1 drop of Vectashield mounting medium (Vector Laboratories, Burlington, Ontario, Canada) and sealed with clear nail polish. Slides were stored at 4 °C until imaging.

### 2.4. Three-Dimensional Quantitative Fluorescent in situ Hybridization (3D Q-FISH) 

Cells grown on slides to 70–80% confluency were permeabilized by incubation in 0.5% Triton X-100 for 10 min. A freeze-thaw treatment was applied. This included soaking the slides in 20% glycerol, then dipping them into liquid nitrogen for a 5–10 s and allowing them to thaw; this procedure was repeated three times. Then, the slides were washed three times in 1× PBS for 5 min before incubating in a 0.1 M HCl solution for 10 min. Hybridization was performed with 5 μL telomeric peptide-nucleic acid (PNA) probe conjugated to a Cy3 fluorophore (DAKO, Glostrup, Denmark). Telomeric probe was applied to the area containing cells, which was covered with an 18 × 18 mm coverslip and sealed with rubber cement. Using a HYBrite Denaturation and Hybridization System (Vysis; Abbott Diagnostics, Des Plains, IL, USA), denaturation was performed at 80 °C for 3 min, followed by probe annealing at 30 °C for 120 min. The slides were then washed in 70% formamide (Fluka; Sigma-Aldrich, St Louis, MO, USA)/10 mM Tris (pH 7.4) twice for 15 min each, followed by a 5-min wash in 0.1× saline sodium citrate buffer (SSC) at 55 °C, and two 5-min washes in 2× SSC/0.05% Tween. DAPI staining was performed with 5 µL of 10 µg/mL DAPI (4, 6-diamidino-2-phenylindole) at room temperature for 3 min in the dark. Excess DAPI was rinsed with water and slides were mounted with 22 × 22 mm coverslips (Zeiss, Toronto, ON, Canada) using a drop of Vectashield mounting medium (Vector Laboratories, Burlington, Ontario, Canada).

### 2.5. 3D-SIM Imaging and Analysis

The cells were imaged using a Zeiss Elyra PS1 SIM a Plan-Apochromat 63x/1.40 Oil immersion objective and captured with an Andor EM-CCD iXon 885 camera with a 1.6X tube lens (all from Carl Zeiss, Canada). The DAPI channel was captured with 405 nm laser excitation, 23 mm diffraction grating and filter cube SR Cube 07. The z-stacks were captured in 91 nm sections with 50–90 z-stacks captured per image. The 3D-SIM and widefield images were reconstructed with ZEN 2012 black edition (Carl Zeiss, Jena, Germany) with the standard settings. Fifty nuclei per treatment were chosen randomly and imaged for each of the three replicates of the experiment. Image processing was performed in MATLAB (MathWorks, Natick, MA, USA). A central z-plane was manually selected and exported as TIF files. The granulometry of the DNA structure and the structure of DNA-poor space was measured with a morphological sieve applied to the error-function clipped images [59]. The coefficient of variation and the skewness of the intensity histogram over the detected region were also calculated. For statistical analyses, the distributions were compared using two-sided, two-sample Kolmogorov–Smirnov (KS) tests to determine any differences. *p*-values of <0.05 were considered statistically significant.

### 2.6. Widefield 3D Imaging and Analysis

Fluorescent images were captured using an AxioImager Z1 microscope with an AxioCamMR3 camera and a Plan-Apochromat 63× Oil DIC M27 lens (all from Carl Zeiss, Canada). Images were captured in 80 z-stacks at 100-nm intervals for assembly of a 3D image of the cell nucleus. The Cy3 channel was imaged with a constant exposure time of 600 ms and the DAPI channel 100 ms. 50 nuclei per treatment were selected for analysis for each of the three replicates in each experiment. Images of selected nuclei were processed using an iterative deconvolution algorithm in the Axiovision software (Carl Zeiss, Jena, Germany), which removes optical distortion and produces a clearer image for analysis. The deconvolved images were exported as TIF files for analysis with the Teloview^TM^ v1.03 software program (Telo Genomics Corp.). Teloview^TM^ was used to assess telomere number, signal intensity distribution, spatial distribution and the presence of telomere aggregates. Statistical analysis for the resulting telomere intensity histograms was performed using a Manzel-Haenszel test with 1 degree of freedom, which tests if the changes between each time-point follow a linear directional pattern.

### 2.7. Metaphase Spread Preparation

Cells were incubated with nocodazole (final concentration: 0.2 µg/mL) (Sigma-Aldrich, St. Louis, MO, USA) in order to arrest them in metaphase. MCF-7 derived cell lines were treated with nocodazole for 4 h prior to harvesting. Cells were harvested into a conical tube, then centrifuged for 10 min at 800 rpm and the media removed. They were then washed in 1× PBS, centrifuged again (800 rpm), and the supernatant removed. The cells were incubated in 5 mL of 0.075 M potassium chloride solution for thirty minutes at room temperature, then centrifuged again and the supernatant removed. Five drops of fixative (3 methanol: 1 acetic acid) were added drop by drop with a Pasteur pipette, waiting a minute in between each drop. Fixative was then added in a dropwise fashion in increasing amounts, with two-minute intervals in between each addition. The cells were allowed to sit at room temperature for 10 min, then re-suspended in the fixative and centrifuged at 800 rpm. The supernatant was removed and the cells re-suspended in 1 mL of fresh fixative, then incubated at room temperature for 20 min. The cells were then centrifuged (800 rpm, 10 min), the supernatant aspirated, and fresh fixative added before storage at 4 °C. For metaphase spread preparation for the MCF7 cell line (both wt and CRISPR-p53), slides were chilled briefly on dry ice until condensation just began forming. The cells were resuspended in fixative and dropped from a height onto the chilled slides. The slides were immediately placed on a 40 °C block warmer for roughly a second and then dipped into 50% glacial acetic acid to fix the spreads. Once the acetic acid had evaporated, the slides were stained with Giemsa’s stain (Gibco^®^ KaryoMAX^®^ Giemsa Stain, Thermo Fisher Scientific, Waltham, MA, USA) for a few minutes. Excess stain was rinsed off with distilled water. The slides were viewed under a 20× objective in order to assess the quality and the quantity of the metaphase spreads present. Acceptable slides were stored at −20 °C until experimentation.

To obtain metaphases from human normal mammary epithelial cells, a modified protocol was used. Briefly, the cells were grown directly on slides, and treated with nocodazole for 10 h when 70–80% confluent. After the incubation with nocodazole, the media was removed, and 5 mL of 0.075 M KCl added and allowed to sit for 12 min. Then, 1 mL of fixative was added gently in a dropwise fashion. The solution was removed, and the slides washed three more times in fixative. They were then allowed to dry at an angle at room temperature, before staining with Giemsa. The slide with the best mitotic spreads was chosen for spectral karyotyping (SKY) experimentation and stored at −20 °C until use.

### 2.8. Spectral Karyotyping (SKY) Slide Preparation and Imaging

Slides selected for spectral karyotyping were first equilibrated in 2× SSC for 10 min, then incubated for 1 h in a 37 °C humidified incubator with 100 uL of 100 ug/mL RNAase in 2× SSC. After washing thrice in 2× SSC for five minutes, the slides were incubated at 37 °C in 50 mL of 0.01M HCl containing 25 uL of pepsin solution. MCF-7 derived cell lines underwent an eight-minute pepsin digestion, while cells from HMECs underwent a 12-min incubation. The slides were washed twice in 1× PBS for five minutes, then in 1× PBS/50 mM MgCl2 for a further five minutes. The slides then underwent a post-fixation in a 1% formaldehyde/1× PBS/50 mM MgCl_2_ solution for ten minutes, before being washed once more in 1× PBS for five minutes. The slides were dehydrated by being passed through an ethanol series of increasing concentration (70, then 80, then 100% ethanol) for three minutes each, then allowed to air dry. Prior to denaturation, the slides were pre-warmed in an oven at 70 °C for five minutes. The slides were incubated in 70% deionized formamide/2× SSC (pH 7.0) at 70 °C for two minutes, then immediately transferred to 70% ethanol (−20 °C), followed by 90% and 100% ethanol (both −20 °C), each for three minutes. The slides were then allowed to air dry. The SKY probe (Spectral Karyotyping Reagent (Human) (Vial #1, ASI kit, Applied Spectral Imaging, Carlsbad, CA, USA) was denatured at 85 °C for five minutes and then maintained at 37 °C in a water bath for 30 min prior to use. 10 uL of the probe was added to each slide, along with 24 × 24 mm coverslips that were sealed with rubber cement. The slides were then placed into a 37 °C humidified incubator for 36 h. After removing the coverslips, the slides were washed thrice in 50% formamide/2× SSC (pH 7.0) at 45 °C for five minutes each, followed by two five-minute washes in 1× SSC at 45 °C. Then, 80 uL of blocking reagent (vial #2, ASI kit, Applied Spectral Imaging, Carlsbad, CA, USA) was added to the slides along with a 24 × 60 mm coverslip, before being returned to the incubator for 30 min. The coverslips were removed, and excess fluid allowed to drain. 5 uL of Cy5 Streptavidin antibody and 5 uL of anti-digoxin antibody were diluted in 4× SSC/1% BSA to a final volume of 1 mL, and 80 uL of the solution applied to each slide. Fresh 24 × 60 mm coverslips were applied, and the slides returned to the incubator for 45 min. Once this incubation was finished, the coverslips were removed and excess fluid allowed to drain, before 80 uL of Cy5.5 Strep Avidin antibody (diluted 1:200 in 4 SSC/1% BSA) was applied to each slide along with fresh coverslips. The slides were again returned to the incubator for 45 min. After removing the coverslips, the slides were washed thrice in 4 × SSC/0.1% Tween 20 at 45 °C for five minutes each time. Excess fluid was allowed to drain, then 50 µL of DAPI (1 ug/mL) was added to each slide along with a 24 × 60 mm coverslip, and incubated in the dark for five minutes. The coverslips were removed and the slides very briefly rinsed in distilled water before mounting with a drop of VECTASHIELD and 24 × 24 mm coverslips.

We used the Spectra Cube™ on a Carl Zeiss Axioplan 2 microscope using a 63× oil objective and the SKYVIEW 1.6.2 and 2.0 softwares. Three independent SKY experiments were performed for each cell line. Results were documented in Microsoft Excel in order to tabulate and statistically analyze cytogenetic changes detected through SKY.

## 3. Results

### 3.1. Analysis of DNA Sstructure Reveals Differences in DNA Structure and Presence of DNA Poor Spaces Between Wild-Type p53 and an Isogenic CRISPR p53 Deleted Cell Line

**Figure 1:**
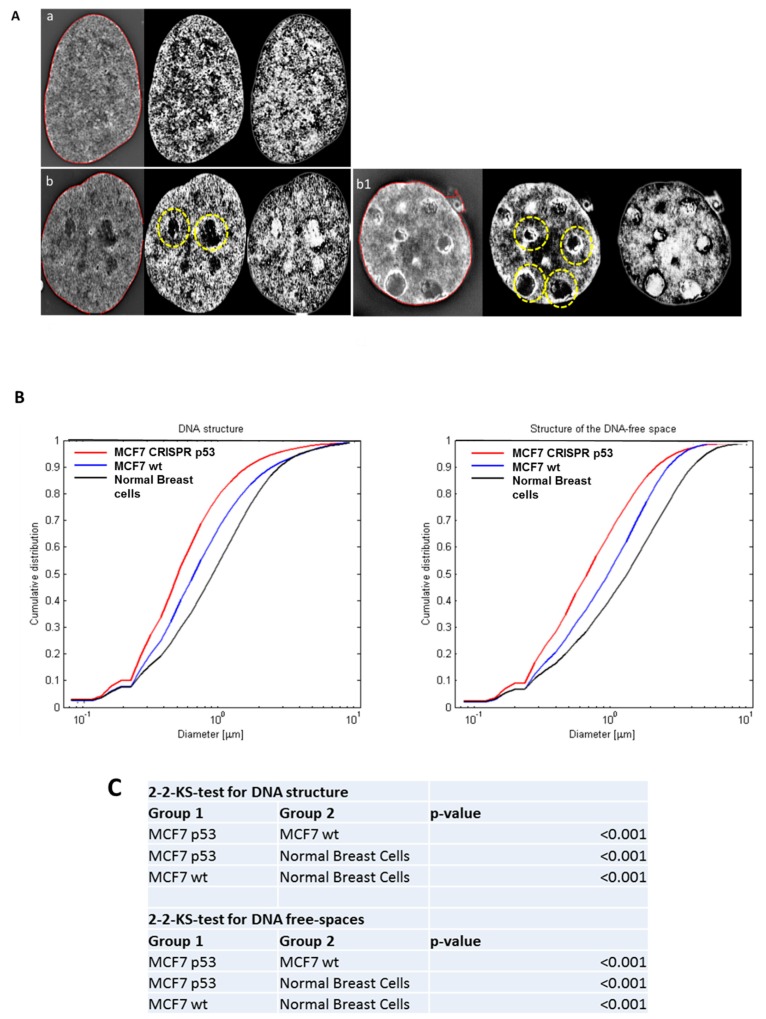
The use of three-dimensional (3D)-SIM (Structured Illumination Microscopy) to investigate differences in DNA structure between normal breast cells, and MCF-7 (Michigan Cancer Foundation-7). (**A**) 3D-SIM images of primary normal breast cells (a), MCF7 wild type (b) and MCF7 CRISPR(Clustered Regularly Interspaced Short Palindromic Repeats) p53 deleted (b1). MCF7 shows more DNA-poor spaces than primary breast cells and CRISPR p53 deleted cell lines shows more DNA-poor spaces than the wild-type ones. Left panels: reconstructed 3D-SIM images; middle panels: light granulometry images; right panels: dark granulometry images. (**B**) Comparisons of granulometry DNA structure and DNA-poor spaces between the three cell lines. (**C**) *p*-value comparisons for the comparisons in (B).

Three-dimensional Structured Illumination Microscopy (3D-SIM) imaging was used to investigate global DNA structure and presence of DNA-poor spaces. 3D-SIM images of DNA structure reveal differences between wild-type (wt) and p53 CRISPR deleted cell lines. In Figure 1, we show a comparison between 3D-SIM images of normal breast cells, MCF-7 wild-type and MCF-7 CRISPR p53 deleted, respectively. Notably, the CRISPR p53 deleted cells show more DNA-free/poor spaces than their normal counterparts (indicated with circles), a characteristic of tumor cells [58,59,60]. From the 3D-SIM images, the different length-scales of DNA organization can be interpreted as granules with different diameters, and the cumulative size distribution of DNA structures can be represented as a curve showing the distribution of different granule sizes in that particular cell [58,59,60]. Histograms for the structure of DNA and structure of DNA-poor spaces for MCF-7 (wt and CRISPR-p53) and normal breast cell lines are shown in Figure 1B. Structures at the low end of this scale range from approximately 200 to 700 nm, and represent the intra-nuclear DNA structures. At the upper end of the scale, sizes from 1–3 μm represent structures closer to the scale of individual nuclei. Therefore, changes at the upper end of the scale indicate shifts in nuclei size. Changes at the lower end of the scale represent shifts in actual intranuclear granule size. Higher granule number at low length scales represents more granularity in the nuclear DNA macrostructure. Granulometry curves demonstrate an increase or decrease in granule size distribution towards small (submicron) structures or large (greater than a micron) structures in the DNA and DNA-poor space between cell populations. Additionally, granulometry curves can display differences in the homogeneity of the granule size distribution. A steeper slope in granulometry means a smaller size distribution, or a more homogenous size distribution. In turn, higher heterogeneity in the size distribution of structures in a given cell population correlates with a less steep curve. Here, we observed an increase in the presence of DNA structures and DNA-poor spaces from wild-type to p53 deleted cell lines. For both DNA structure and structure of DNA-poor space, the wild-type cell lines show significantly less submicron DNA structure than their p53-deficient counterparts. A similar difference in DNA structure between malignant and non-transformed cells has been noted between tumor and control cells [60,61,62]. These data along with the data presented here help to demonstrate the fundamental changes in nuclear structure between transformed and non-transformed cells. 

Figure 1 exhibits the use of 3D-SIM to investigate differences in DNA structure between normal breast cells and MCF-7. In panel A, we show a representative 3D-SIM image of normal primary breast cell nuclei (a), MCF7 wild type (b), MCF7 CRISPR p53 deleted (b1). It is clear that MCF7 shows more DNA-poor spaces than primary normal breast cell nuclei, while CRISPR p53 deleted cell line show more DNA-poor spaces than the wild-type ones. In panel B, we quantitatively compare the amounts of DNA structure and DNA-poor spaces between the cell lines using granulometry. The results of comparisons between cell lines and accompanying p-values are show in panel C.

### 3.2. Analysis of 3D Nuclear Telomere Organization Shows Increase of Genomic Instability between Wild-Type p53 and Isogenic CRISPR p53 Deleted Cell Line

**Figure 2:**
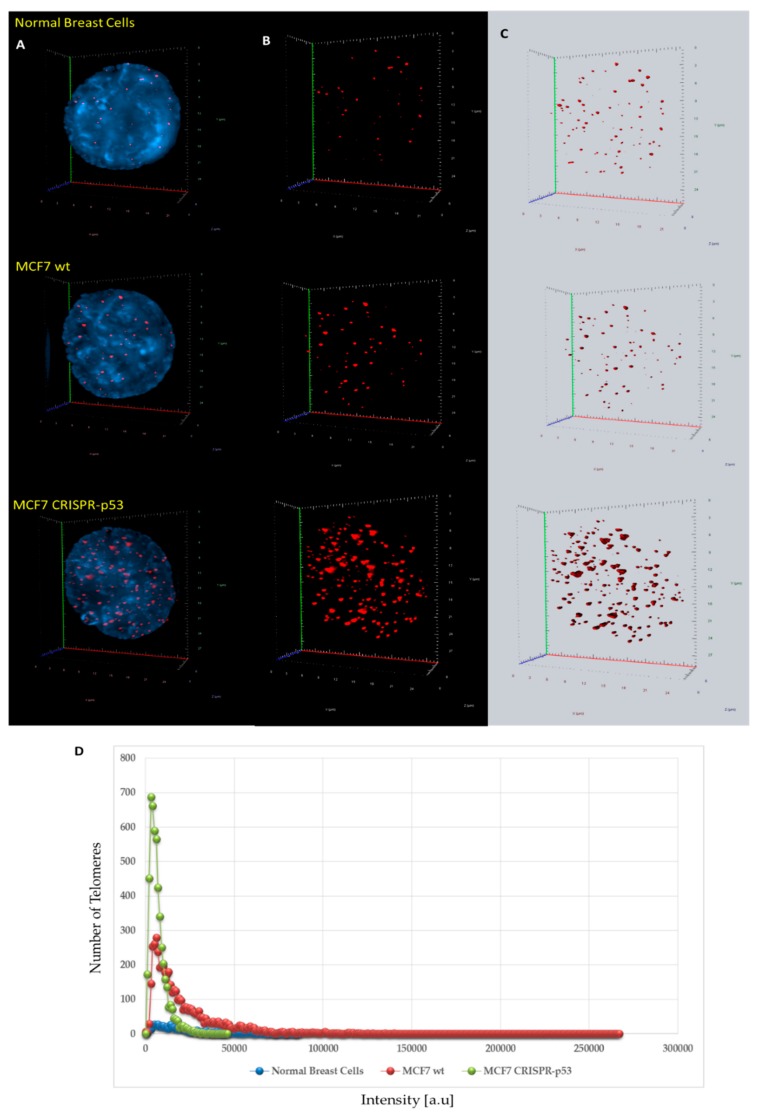
Differences in 3D telomere distribution between normal breast cells and p53 knockout and wild-type cells in isogenic MCF7 cell lines. (**A**–**C**) Representative nuclei, counterstained with DAPI (4′,6-diamidino-2-phenylindole) (blue) from normal breast cells, MCF7 wild-type and MCF7 CRISPR-p53 deleted, where Cy-3 labelled telomeres appear as red dots. (**D**) A telomere intensity histogram showing distribution of signal intensities in normal breast cells and MCF7s (wt and p53 knockout). Numerous parameters were altered between the three cell lines. Most notably, in the MCF7 CRISPR-p53, compared to the isogenic wild-type, there was a dominance of shorter telomeres, which by itself is indicative of telomere dysfunction and genomic instability. [a.u.]—arbitrary units. Abscissa = intensity [a.u]; ordinate = number of telomere signals.

**Figure 3:**
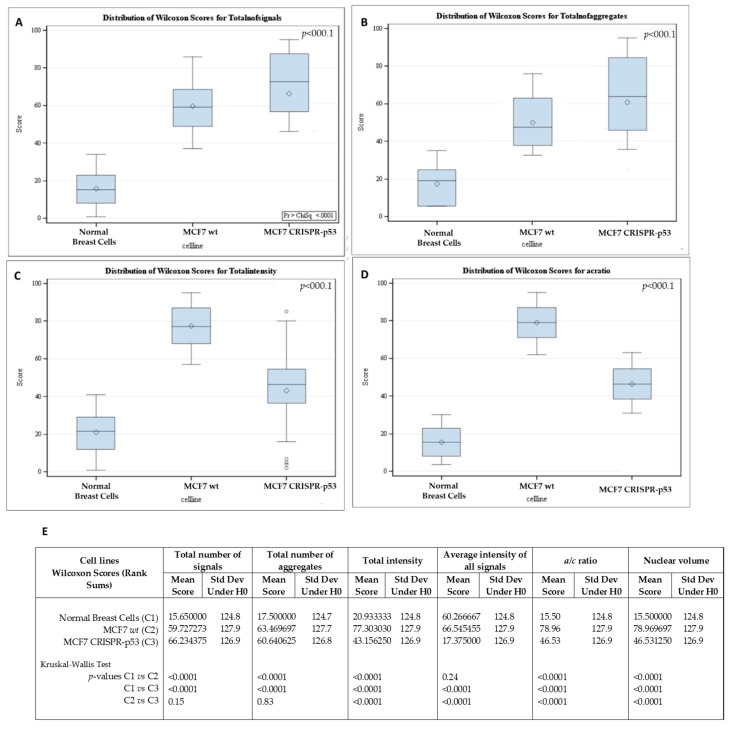
Differences in telomere parameters between normal breast cells, p53 knockout and isogenic wild-type MCF-7 cells. (**A**) The total number of telomere signals. (**B**) The total number of telomere aggregates (telomeres in close proximity that cannot be further resolved at an optical resolution limit of 200 nm). (**C**) Total telomere signal intensity (proportional of telomere length). (**D**) *a/c* ratio (nuclear spatial distribution of telomeres). The *a/c* ratio is defined as the nuclear space occupied by telomeres, represented by three axes of length *a*, *b* and *c*. The ratio between the *a* and *c* axes, the a/c ratio, reflects the distribution of telomeres, which changes at different stages of the cell cycle. (**E**) *p*-values for each comparison. Std Dev—standard deviation; H0—null hypothesis.

3D telomeric quantitative fluorescent in situ hybridization (3D Q-FISH) was used to investigate changes in the nuclear architecture of telomeres. MCF7 (wt and CRISPR-p53) and normal breast cells were compared in their nuclear architecture of telomeres using TeloView^TM^. Comparisons between wild-type and p53 knockout MCF7 cells are shown in Figure 2 and Figure 3. Signal intensity histograms reveal a significant increase of short telomeres in the p53 knockout cells when compared with the p53 wild-type cells (*p* < 0.001) (Figure 2D). In Figure 2D, we plot telomere length (signal intensity in arbitrary units) on the x-axis against the number of telomeres on the y-axis. The signals with the same intensity fall on the same region of the graph, providing the distribution of telomere length in each cell line. For the normal breast cells, for example, this plot has a single peak, ranging from 20 to 40 telomeres per nucleus on the y-axis. Interestingly, the number of telomere signals and the formation of telomere aggregates increase in the p53 knockout MCF7 (Figure 3). In the MCF7 cells, the total intensity (telomere length) and the a/c ratio decrease after p53 deletion. This suggests that critical shortening of the telomeric repeats led to dysfunctional telomeres and fusions (telomere aggregates). Resulting dicentric chromosomes can initiate ongoing chromosomal instability via breakage–bridge–fusion cycles where breaks constantly generate telomere-free ends, decreasing total intensity and leading to overall genetic changes that contribute to genomic instability. This ongoing genomic instability decreases the proliferation rate of the MCF7 p53 knockout cells, as indicated by the decreased a/c ratio in p53-deficient MCF7 cells compared to their wt counterparts (*p* < 0.0001). The a/c ratio represents the nuclear space occupied by telomeres and gives some indication of the cell cycle phase (G0/G1, S, G2) [50].

### 3.3. p53 CRISPR Deletion Increase Chromossomal Instability in MCF7

**Figure 4:**
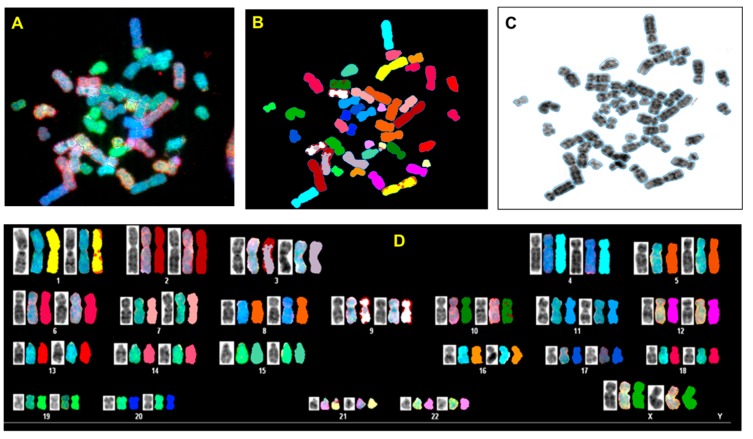
Spectral karyotyping (SKY) of representative metaphase from normal breast cell (HMECs). The SKY was performed as described (Materials and methods). (**A**) metaphase spread: raw image; (**B**) Metaphase spread: spectral image; (**C**) metaphase: inverted DAPI image, (**D**) classified spectral karyotype of the identical metaphase. SKY was performed in three independent experiments as described in the Materials and Methods. A minimum of 20 metaphases was analyzed per experiment.

**Figure 5:**
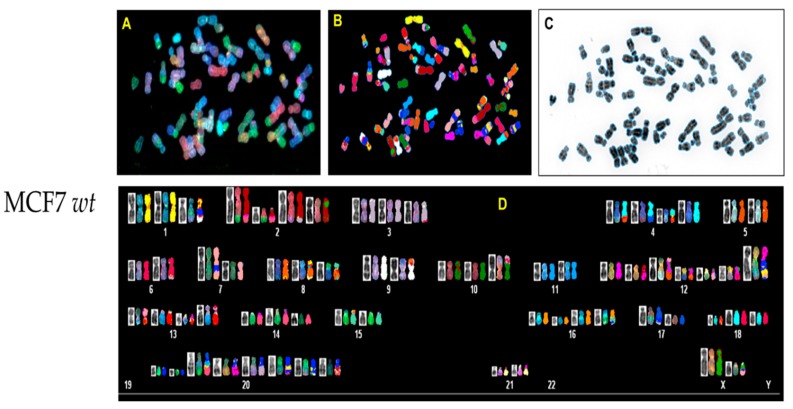

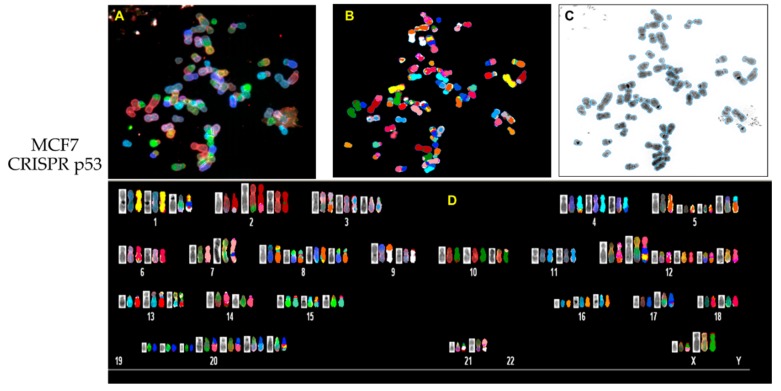
Spectral karyotyping (SKY) of representative metaphase from MCF7 wt and CRISPR p53 deleted cell lines. SKY was performed as described (Materials and Methods). (**A**) Metaphase spread: raw image; (**B**) metaphase spread: spectral image; (**C**) metaphase: inverted DAPI image, (**D**) classified spectral karyotype of the identical metaphase. SKY was performed in three independent experiments as described in Materials and methods. A minimum of 20 metaphases was analyzed per experiment. Upper panel: MCF7 wt; lower panel: MCF7 p53 deleted.

In order to analyze possible chromosomal abnormalities after deletion of TP53, we applied spectral karyotyping (SKY) analysis to metaphase chromosome preparations. The SKY technique results in the simultaneous color discrimination of all 24 human chromosomes [63,64]. As shown in representative images in Figure 4 and Figure 5, p53 CRISPR deleted cells show more numerical chromosome gains as well more as chromosome aberrations, including insertions, deletions or fusions. In Table S1, we show a detailed overview of the chromosomal aberrations detected by SKY in all cell lines.

### 3.4. Nutlin-3 Affects Telomeric Nuclear Architecture But Not Super Resolution DNA Structure

**Figure 6:**
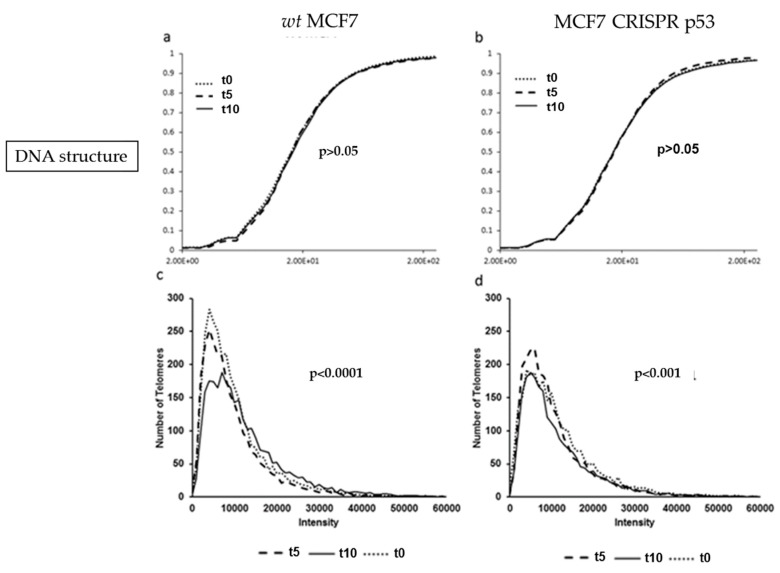
Comparison of DNA structure (using granulometry) and telomere histograms between the MCF7 (wild-type and CRISPR-p53) after 0, 5 or 10 h of Nutlin-3 treatment. (**a**) and (**b**) show the cumulative distribution of DNA structure. (**c**) and (**d**) show the telomere length (signal intensity in arbitrary units) on the x-axis against the number of telomeres on the y-axis. The *p*-values demonstrate the comparison between t0 or t5 with t10.

Wild-type and p53 knockouts of MCF7 cell lines were incubated with 10 uM of the p53 re-activating compound Nutlin-3, and harvested at 0, 5 and 10 h post-treatment. Nutlin-3 binds to MDM2 and prevents binding to p53, thereby upregulating p53 protein levels [22]. MCF7 cells treated with Nutlin-3 did not show significant change in DNA structure or DNA poor spaces (data not shown) after 10 h post treatment (*p* > 0.05), as seen in Figure 6a. Similarly, no significant change in DNA structure was observed in the p53 knockout MCF7 cells after Nutlin-3 incubation after 10 h post-treatment (*p* > 0.05), shown in Figure 6b. However, the treatment with Nutlin-3 decreased the peak of short telomeres in MCF7 wt but not in the isogenic CRISPR-p53 deleted MCF7 cells.

### 3.5. RITA affects nuclear DNA structure

**Figure 7:**
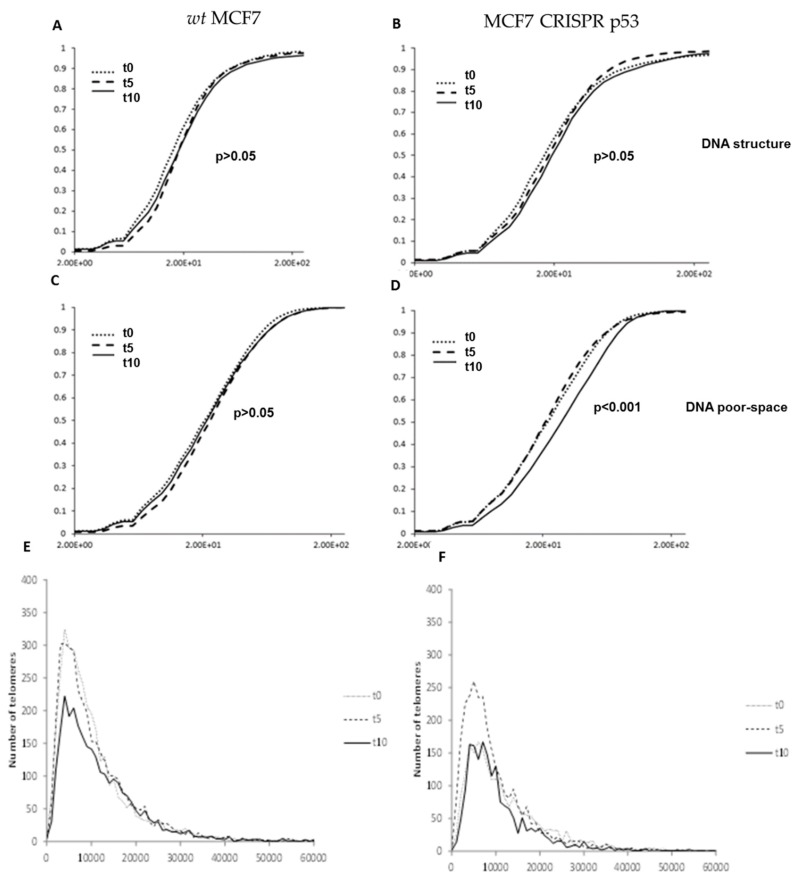
Changes produced by RITA in nuclear architecture in both wild-type and p53 knockout isogenic MCF-7 lines. Granulometry curves of DNA structure of wild-type MCF-7 (**A**) and p53 knockout MCF-7 cells (**B**). Granulometry curves of the structure of DNA-poor space of wild-type (**C**) and p53 knockout MCF-7 cells (**D**). Differences in 3D telomere distribution between p53 knockout and wild-type cells lines after RITA treatment (E).

Wild-type and p53 knockout MCF7 cells were also treated 10 uM of RITA and harvested at 0, 5 and 10 h treatment durations. RITA binds to wild-type p53 and prevents MDM2 from binding, which stabilizes p53 protein levels [23]. Granulometry curves of DNA structure and DNA-poor space for wild-type MCF7 cells treated with the p53 stabilizing substance RITA are shown in Figure 7. These data show significant response at 10h post treatment in decreasing DNA-poor spaces in the MCF7 CRISPR-p53 (*p* < 0.001, Figure 7D). Signal telomere intensity histograms for MCF7 wild-type and CRISPR p53 cell lines after treatment with RITA were also calculated. There were significant changes in telomere intensity histograms over time. For example, as seen in Figure 7E,F, we observed a sharp decrease in low intensity telomere signals at 10 h post-treatment. This effect may indicate that the response to RITA is distinctly time-sensitive in these cells, as evidenced by the granulometry data. Effects such as cell density may produce a substantial change in the timing of this response. Interestingly, RITA seems to show an effect on MCF7 cells regardless of p53 status.

## 4. Discussion

Although the tumor suppressor p53 was one of the first to be characterized as a transcription factor, there are still many unresolved questions about its mechanism of action [53,65,66,67,68]. One important question is whether the p53 transcriptional program can affect chromatin architecture. Our results in 3D-SIM showed a significant difference in DNA structure and DNA-poor spaces in MCF7 cells after p53 deletion. As previously observed, transformed cells exhibit a greater range of granule sizes (more granularity) when compared to non-transformed cells, which exhibit a smaller range of granule sizes on average [59,60]. This manifests in the image as a much “coarser” texture of DNA structure in malignant cells compared with the “fine” texture of DNA structure observed in non-transformed cells. This difference in granularity likely reflects dysfunctions in cellular mechanisms, which normally organize chromatin into higher order structures. The organization of chromatin in domains is mediated by a diverse group of proteins which bind and cross-link DNA establishing functionally distinct compartments [69], known to be dysregulated in a variety of cancers [70]. For example, many cancers exhibit defects in the expression of non-histone DNA binding proteins such as the high mobility group proteins HMG-I and HMG-Y [71]. These proteins have been shown to regulate chromatin organization by cross-linking DNA fibers and inducing heterochromatin clustering [72]. Thus, establishing functionally distinct chromatin compartments in this manner correlates with changes in gene expression and the overexpression of these proteins is linked to neoplastic transformation [73,74,75]. As such, we can interpret changes in the granularity of DNA structure as reflective of the dysfunction in chromatin domains observed in cancer cells, which contributes to abnormal gene expression, genomic instability and altered cell behavior characteristic of malignant cells. One limitation of this study is that no additional staining using anti-nucleolin antibody or upstream-binding factor (UBF) to differentiate what we call circular “DNA poor spaces” from nucleoli was performed. The granulometry program measures DNA content and absence of DNA signals and therefore does not distinguish nucleoli from DNA poor spaces. Thus, the increase of DNA-poor spaces that we observed in the p53-CRISPR deleted cell lines compared with the wild-type ones could be also due to hypertrophy of nucleoli, rather than increase of DNA-poor spaces or due to both. The increase in the size and number of nucleoli has been correlated with an enhanced rate of cell proliferation and growth in cancer tissues [76].

The 3D telomere analysis revealed key differences in the nuclear architecture between the wild-type and p53 knockout MCF7 cell lines. We observed that p53 deletion increased genomic and chromosomal instability, represented by an increase in telomere signals, telomere aggregates and chromosomal rearrangements. We also observed a decrease of total telomere intensity (proportional to length). Although we found a significant increase in telomeric instability after p53 deletion in MCF7 cells (shorter telomeres and increase of telomere aggregates), the accumulation of chromosomal abnormalities at this point was not as high as we expected. Therefore, our data suggest a model in which telomere shortening occurs in the early stage of carcinogenesis (after TP53 deletion), leading to an increase in the number of telomeric end-to-end fusions (increased telomere aggregates), eventually giving rise to dicentric chromosomes and chromosomal instability only at a later stage.

Re-activation of p53 with the compound Nutlin-3 resulted in significant changes in 3D architecture as revealed by 3D telomere analysis in MCF7 wt. However, changes in DNA structure and DNA-poor spaces were not observed with 3D-SIM even after 10 h of Nutlin-3 treatment. Higher concentrations as well as longer time points may be necessary to detect significant changes. Treatment with RITA also resulted in changes in telomeric nuclear architecture. Data from granulometry measurements indicated that RITA treatment led to decreases in the DNA-poor spaces areas in the MCF7 p53 CRISPR-deleted cell line. Further studies should provide a more precise dissection of RITA-induced changes in DNA structure, but it is possible that RITA exerts an apoptotic effect, which is known to cause changes in nuclear architecture [77].

Results from both 3D-SIM experiments and 3D telomere Q-FISH experiments show that the p53 reactivating compound RITA had an effect even in p53-knockout cells. By contrast, the effect of compound Nutlin-3 on nuclear architecture appeared to be p53 dependent: no significant changes were observed in the corresponding p53 knockout cells. These results indicate a potential non-p53 dependent mechanism of RITA activity. Previous reports attributed RITA activity as being p53-dependent, based on the fact that treatment with pifithrin-alpha, a compound known to block p53 transactivation of target genes, prevented the effects of RITA [78]. However, another study comparing RITA treatment in 14 cell lines showed induction of apoptosis not only in wild-type p53 tumor cell lines, but also in p53-mutant and p53-null cells, whereas nultin-3 was strictly p53-dependent [25]. Additionally, RITA treatment resulted in upregulation of some p53 target genes independently of p53 status. Further analysis showed RITA-dependent activation of p38 and the JNK/SAPK pathway in p53-null cells that resulted in mitochondrial apoptosis [25]. RITA has also been shown to be effective in a multiple myeloma cells independently of p53 [79,80] and in cell types expressing mutant p53 protein [81]. These results are in line with our observations that RITA can induce architectural remodeling independently of p53 status in cancer cells [82].

Indeed, we observed different effects of the compounds Nutlin-3 and RITA on cells, consistent with recent data, which indicate that Nutlin-3 and RITA can behave differently even though both target the p53--MDM2 regulatory pathway [24]. This may be attributed to other cellular effects RITA has been implicated in, including effects involving the p38 and the JNK/SAPK pathways [25]. Additionally, RITA-bound p53 may have altered activity compared with wild-type p53. p53 is highly sensitive to post-translational modifications which can profoundly modulate the specific activities of this gene [83]. It is also known that conformational changes in p53 can affect DNA binding and transactivation activity, which is often seen in tumors expressing mutant forms of p53 [81]. The differential effects of RITA-stabilized p53 and Nutlin-3-stabilized p53 have been demonstrated directly through differences in gene expression profiles between the two, with RITA-bound p53 showing a preference for transcriptional programs leading to apoptosis rather than growth arrest [24,83,84]. As such, the differential effects of RITA and Nutlin-3 as observed in the present report lend further support to the divergent nature of their individual roles.

One question that naturally arises is whether this p53-dependent effect can be seen in non-transformed cells, or whether it only occurs in tumor cells. Supporting this argument is the evidence that RITA is pro-apoptotic in tumor cells but not in untransformed cells [23]. Even though the investigation of the effects of RITA and Nutlin-3 in primary cells was not part of this study, this should be addressed in future studies, in order to assess whether the effects of p53 expression on nuclear architecture, with and without RITA and Nutlin-3 treatment, are dependent on neoplastic transformation.

In conclusion, we investigated the role of tumor suppressor gene p53 and its relation to one of the hallmarks of cancer: dysregulated nuclear architecture. In this model, we used breast cancer cells to see if remodeling of DNA structure and telomere nuclear architecture could be induced upon pharmacological reactivation of p53 activity. Our observations indicate a significant difference in nuclear architecture of wild-type and p53 knockout tumor cells in vitro as revealed by super resolution analysis of DNA structure and 3D nuclear telomeric analysis. We also found that Nutlin-3 induced architectural remodeling in tumor cells in a p53- dependent manner, while RITA produced significant changes in structure of DNA poor spaces, independently of p53 status. These data provide valuable insight into the role of p53 in regulating nuclear architecture, which might be involved in tumor suppression by p53. Greater understanding of the mechanisms of p53-dependent anti-cancer activity may help pave the way for developing more efficient cancer therapies. *TP53* represents an important target for anticancer drug discovery. Many compounds have been developed to target p53 and its regulators with an overall goal to either activate p53 in cancer cells or to inactivate p53 temporarily in normal cells for chemoradiation protection. However, effective personalized cancer therapy requires also the monitoring of the efficiency of those compounds alone or in combination with other cancer therapy strategies. Therapy response monitoring using imaging methods is an important component of personalized/precision medicine. 3D telomere signatures together with 3D-SIM could be used to guide changes in therapy in order to improve the efficacy of the treatment. Both technologies are minimally invasive and could identify ineffective therapies shortly, instead of waiting for months until treatment failure becomes apparent.

## Supplementary Materials

The following are available online at https://www.mdpi.com/2077-0383/9/2/598/s1, Figure S1: Validation of p53 knockout by western blot analysis, Table S1: Chromosomal aberrations in normal breast cell and MCF7 (wt and CRISPR p53).
