# Supplementary figures and images for: p53 CRISPR Deletion Affects DNA Structure and Nuclear Architecture
Source: J Clin Med. 2020 Feb 22;9(2):598. doi: 10.3390/jcm9020598 (PMC7073688; doi:10.3390/jcm9020598)

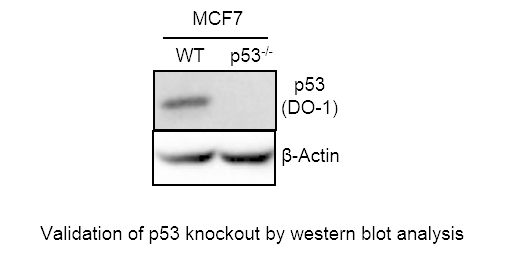

Supplement: Supplementary file 1 [file jcm-09-00598-s001.zip › jcm-694845 supplementary/Supplementary Figure S 1.jpg]
